# Postprandial exercise attenuates glucose and insulin and is associated with age, cognitive function, and extracellular/intracellular body water

**DOI:** 10.14814/phy2.70657

**Published:** 2025-11-24

**Authors:** Michael A. Petrie, Kristin A. Johnson, Patrick M. McCue, Akanksha Aggarwal, Tristan Brown, Katrina Grevengoed, Jordan Hoyman, Ali Kallner, Jack Klein, McKenna Lloyd, Michelle Patterson, Richard K. Shields

**Affiliations:** ^1^ Department of Physical Therapy and Rehabilitation Science, Carver College of Medicine The University of Iowa Iowa City Iowa USA; ^2^ Department of Health and Exercise Science Colorado State University Fort Collins Colorado USA; ^3^ Present address: Department of Internal Medicine University of Pittsburgh Pittsburgh Pennsylvania USA; ^4^ Present address: OrthoArizona‐North Peoria Peoria Arizona USA; ^5^ Present address: Le Mars Physical Therapy Le Mars Iowa USA; ^6^ Present address: Unity Point Health Hiawatha Iowa USA; ^7^ Present address: Denver Physical Therapy at Home Denver Colorado USA; ^8^ Present address: Summit Orthopedics Vadnais Heights Minnesota USA; ^9^ Present address: Panorama Physical Therapy Highlands Ranch Colorado USA; ^10^ Present address: Boone County Hospital Boone Iowa USA

**Keywords:** body composition, metabolism, physical activity, precision rehabilitation

## Abstract

Postprandial exercise attenuates glucose/insulin and is recommended for people with metabolic disease. The magnitude of the attenuation has not been directly compared in healthy older and younger people, who are active exercisers, or correlated to biomarkers associated with aging like body water ratios and cognitive function. We determined whether a dose of prescribed exercise attenuated postprandial insulin and glucose in younger and older participants; then, we determined if age, cognitive function, and extracellular to intracellular body water levels (ECW/ICW) were associated with that attenuation. Thirty‐six people, 19 younger adults (seven female) and 17 older adults (nine female), had blood samples taken after a standardized meal followed by exercise or quiet sitting. Participants completed the National Institutes of Health Toolbox assessing cognitive function and had their anthropometrics assessed including ECW/ICW. Postprandial exercise decreased insulin and glucose in younger and older adults compared to postprandial sitting (*p* < 0.001, *p* = 0.003). There were strong correlations between the extent of the metabolic response after exercise and age, ECW/ICW, and cognitive function. Our results highlight that postprandial exercise reduced insulin and glucose among young and old active exercisers and this change was associated with a person's cognitive function and body water compartment ratio (ECW/ICW).

## 1 INTRODUCTION

Sedentary lifestyles yield an imbalance in daily caloric intake relative to daily energy expenditure leading to weight gain (Sharma & Padwal, 2010) with documented implications on health and well‐being (Arsenault et al., 2007; Berrington de Gonzalez et al., 2010; Jin et al., 2023; Zhang et al., 2015). Metabolic dysfunction (Amati et al., 2009; Bankoski et al., 2011; Hansen et al., 2012; Hu et al., 2004; Lee et al., 2012; van der Ploeg et al., 2012) is related to cardiovascular disease (Lee et al., 2012; Patterson et al., 2018; van der Ploeg et al., 2012) and ultimately leads to cognitive decline and dementia (Arvanitakis et al., 2018; Boccara et al., 2023; Geng et al., 2018). Chronic elevation of insulin (hyperinsulinemia) may be a precursor to insulin receptor sensitivity loss and metabolic disease (Janssen, 2021). Therefore, timely physical activity may represent an important modifiable behavior to assist in preventing the persistent rise in global metabolic disease.

Sedentary behavior, especially following a meal, may represent a lifestyle behavior that contributes to the development of type 2 diabetes and other related chronic diseases (Bankoski et al., 2011; Hu et al., 2004; Katzmarzyk et al., 2009; Matthews et al., 2012; Regensteiner et al., 1991). There are also links between diabetes and mild cognitive impairment and dementia with age (Boccara et al., 2023; Low et al., 2021; Pedersen et al., 2012). Insulin, the hormone responsible for lowering blood glucose concentration, is secreted after a meal to lower postprandial glucose elevation. Postprandial hyperinsulinemia is associated with a sedentary lifestyle and may contribute to metabolic dysfunction and insulin resistance (Janssen, 2021). Postprandial hyperinsulinemia has been reported to be attenuated with postprandial physical activity (Bellini, Nicolo, Bazzucchi, & Sacchetti, 2022; Bellini, Nicolo, Rocchi, et al., 2022; Engeroff et al., 2023; Gale et al., 2023) via an “insulin independent” transport mechanism whereby a contracting muscle activates the AMP‐activated protein kinase (AMPK) pathway that turns on Glucose transporter type 4 (GLUT‐4) to move glucose into the muscle (Goodyear & Kahn, 1998; Kjobsted et al., 2017; Ryder et al., 2001). If this mechanism is insulin independent, then healthy older and younger people may not show a differential response even if they have different levels of insulin sensitivity. If active exercisers have saturated the insulin receptor sensitivity capacity, then perhaps active exercisers will get a limited attenuation with exercise after a meal. To our knowledge, no previous study has directly examined the postprandial glucose and insulin response among active, healthy, younger and older people following a low dose of exercise using the same protocol. A recent meta‐analysis offers sound evidence that hyperinsulinemia after a meal may be attenuated with various forms of physical activity (Loh et al., 2020), but no head‐to‐head assessment of chronically active older and younger participants was identified. Health care providers today do not routinely recommend postprandial activity for people with glucose control issues (Petrie et al., 2023), much less differentially assess the benefits for active older and younger people.

Links between obesity, systemic metabolic disease, and brain rescue programs for cognitive impairment have recently gained attention (Arvanitakis et al., 2018; Boccara et al., 2023; Geng et al., 2018; Lee & Shields, 2022; Pedersen et al., 2012). Progression from mild‐to‐severe cognitive impairment is associated with a reduced capacity for brain cells to utilize glucose (Bellini, Nicolo, Bazzucchi, & Sacchetti, 2022), hypertension (Bellini, Nicolo, Bazzucchi, & Sacchetti, 2022), and the accumulation of excessive extracellular body water (ECW) relative to total or intracellular body water (ICW) (Cole et al., 2021; Lee & Shields, 2022; Low et al., 2021; Oliveira et al., 2022; Seo et al., 2013). The ratio of ECW to ICW may provide a unique noninvasive biomarker for systemic metabolic health and is associated with cognitive performance during aging (Seo et al., 2013). Interestingly, glycogen levels increase the ICW and lower the ECW/ICW ratio in skeletal muscle (Shiose et al., 2016). A connection between glycogen and glucose uptake has been intimated and may be associated with postprandial exercise and glucose transport in skeletal muscle. Accordingly, we sought to explore if the ability to attenuate the postprandial glucose and insulin response with exercise would be univariately associated with the distribution of body water and cognitive performance. To achieve our goals, we designed a study to assess the acute postprandial metabolic response to exercise versus a sedentary condition, and then secondarily explore univariate relationships between postprandial metabolic responses (glucose and insulin), body water compartment ratios, and cognitive performance.

The primary purpose of this study was to determine if postprandial glucose and insulin are attenuated with a specified dose of exercise in active healthy younger and older people. The secondary purposes of this study are to determine if fasting glucose and insulin are associated with ECW/ICW and cognitive function, and lastly, to determine if the change in glucose and insulin after postprandial exercise is associated with ECW/ICW and cognitive function. We expect that postprandial insulin, and glucose will decrease after a dose of exercise in both older and younger participants with a greater decrease in the older participants. We also expect that there will be novel univariate associations among changes in glucose, insulin, ECW/ICW, and cognitive performance.

## 2 METHODS

### 2.1 Participants

Thirty‐six people (19 younger adults (seven female) and 17 older adults (nine female)) participated in a 2‐week study to determine the influence of postprandial low‐intensity exercise. A summary of participant demographics is shown in Table 1. Participants were separated into a cohort of younger adults (26.2 ± 5.1 years) and older adults (70.7 ± 6.4 years). All participants indicated they were healthy and able to walk independently without pain. People with a history of severe cardiovascular or metabolic disease were excluded from participation. People who were receiving hypertension or cholesterol medication controlled with pharmaceutical prescriptions were permitted to participate (*N* = 2). Additionally, participants with non‐pharmacologic management of prediabetes were permitted to participate (*N* = 1), but those using pharmaceutical T2DM management were excluded from participation. All participants provided written informed consent approved by the University of Iowa institutional review board in accordance with the Declaration of Helsinki.

**TABLE 1:** Participant demographics, anthropometric, physical activity assessments, and baseline measurements.

	Younger adult (mean ± standard deviation)	Older adult (mean ± standard deviation)	*p* Value
Age (years)^a^	26.2 ± 5.1	70.7 ± 6.4	*p* < 0.001
Age‐predicted max heart rate (bpm)^a^	194 ± 5.1	149 ± 6.4	*p* < 0.001
Height (cm)	175 ± 7.1	172 ± 10.5	*p* = 0.18
Weight (kg)	72.2 ± 10.7	72.9 ± 10.3	*p* = 0.79
Body mass index (BMI)	23.6 ± 2.3	24.6 ± 2.6	*p* = 0.239
Body fat (%)^a^	20.3% ± 6.3%	28.2% ± 8.6%	*p* < 0.001
Fat‐free mass (kg)^a^	57.8 ± 11.0	52.5 ± 10.6	*p* = 0.04
Basal metabolic rate (BMR)^a^	1686 ± 230	1574 ± 222	*p* = 0.04
Physical activity level (PAL)^a^	2.0 ± 0.2	2.3 ± 0.4	*p* < 0.001
ECW/ICW ratio^a^	0.59 ± 0.01	0.62 ± 0.02	*p* < 0.001
Estimated daily calories	3231 ± 681	3540 ± 757	*p* = 0.123
# Clif bars calculated	3.2 ± 0.7	3.6 ± 0.8	*p* = 0.123
Fasting venous glucose (mg/dL)^a^	89.5 ± 8.1	98.1 ± 5.8	*p* < 0.001
Fasting venous insulin (μUI/L)	6.8 ± 3.5	8.1 ± 2.7	*p* = 0.091
Fasting QUICKI^a^	0.37 ± 0.02	0.35 ± 0.02	*p* = 0.019
HOMA‐IR^a^	1.5 ± 0.7	2.0 ± 0.7	*p* = 0.04
Fasting venous glucose‐insulin ratio	16.0 ± 5.9	14.0 ± 4.3	*p* = 0.111
DCCS cognitive assessment^a^	116.4 ± 5.6	106.8 ± 8.2	*p* = 0.002
Flanker cognitive assessment^a^	111.3 ± 4.1	98.9 ± 6.4	*p* < 0.001
Pattern comparison^a^	143 ± 12	101 ± 16	*p* < 0.001
List sorting working memory^a^	116 ± 9	102 ± 9	*p* < 0.001

Abbreviation: ECW, extracellular water; ICW, intracellular water.

^a^ Indicates significant (*p* < 0.05) different using a studentized *t*‐test by age group (younger adult vs. older adult).

### 2.2 Study design

Each participant completed three sessions on separate days. Session 1 involved taking a fasting venous blood sample, completing a cognitive assessment, and having anthropometrics assessed. In sessions 2 and 3 participants consumed a meal and either exercised or remained sedentary (randomized in session 2; opposite protocol in session 3). Each participant received a blood draw 30 min after the meal in sessions 2 and 3. During the sedentary sessions, participants were asked to passively sit in a chair for the duration of the study visit, while during the exercise sessions participants were asked to complete a 15‐min walk at a mild–moderate pace after the meal (60% of their age‐adjusted maximum heart rate). A Rating of Perceived Exertion Scale was used during the exercise with a target of 4 on the 10‐point scale. The order of exercise versus sedentary condition in session 2 was randomly selected for every other participant so that the order of testing exercise and sedentary was counter‐balanced. Participants were permitted to continue with their regular activity routine, but were asked to refrain from any light, moderate, or high‐intensity exercise 24 h before each study session. Participant diet was not controlled during the separation between sessions, but participants were asked to maintain a similar level of activity and food intake over the duration of the study. Lastly, all participants were required to attend each study session after having completed an overnight fast (at least 6 h of no food), but were reminded to stay hydrated.

### 2.3 Anthropometric and physical activity assessments

In session 1, all participants completed a series of anthropometric measurements that included height, weight, body composition, and water compartment levels. General body composition was measured using a bioelectric impedance analysis system (InBody S10, InBody Co., Seoul, South Korea). The bioelectric impedance analysis provides estimates of multiple body composition metrics, including fat‐free mass, intracellular water, and extracellular water. Participants completed a physical activity level questionnaire and the International Physical Activity Questionnaire (7‐Day Long‐Form) to assess their regular physical activity levels. Additionally, the physical activity level questionnaire helped determine the target meal size based on the number of calories consumed each day and their basal metabolic rate, obtained from the impedance analysis.

### 2.4 Standardized meal protocol

The standardized meal consisted of protein bars (Chocolate Chip Clif Bar; Clif Inc., Emeryville, CA, USA). Each participant was asked to consume approximately 25% of their daily energy expenditure estimated from their basal metabolic rate and a self‐reported physical activity level questionnaire. Daily caloric expenditure was calculated by multiplying their total physical activity level, as self‐reported, by the estimated basal metabolic rate (BMR). BMR was calculated by multiplying the participant's fat‐free mass (in kilograms) by 20.9 then adding 478 as described by Ravussin et al. (Ravussin et al., 1986). The estimate for fat‐free mass was obtained from the bioimpedance assessment. Five older participants were uncomfortable consuming the target mixed meal size in 30 min; therefore, the consumption was documented and held constant for the follow‐up session (exercise or sedentary). After consumption of the standardized meal, participants stayed seated for 15 min. Thereafter, participants in the rest session remained seated for an additional 15 min, while the participants in the exercise session performed the brisk walk exercise for 15 min. Each participant experienced both sessions. Thirty minutes after consumption of the mixed meal, participants had a venous blood draw.

### 2.5 Blood collection and analysis

Venous blood was obtained from the forearm with an aseptic venipuncture technique. Venous blood was collected in 5 mL serum vacutainers (Part #: 367814, Becton, Dickenson Co. Franklin Lakes, NJ, USA; RRID:SCR_008418). The collected blood sample was allowed to clot before being centrifuged at 3000 G for 15 min to separate serum. Serum glucose (12‐G7517‐160, HORIBA Instruments Inc., Canton, MI, USA), insulin (KAI‐040, Kamiya Biomedical Company, Seattle, WA, USA; RRID:SCR_013444), and lactate (L7596, HORIBA Instruments Inc., Canton, MI, USA) were quantified using an automated clinical blood chemistry analyzer (BS200, Mindray Bio‐Medical Electronics Co., Shenzhen, CN; RRID:SCR_026779), according to the manufacturer's recommendations.

We calculated fasting glucose/insulin ratios, HOMA‐IR (Matthews et al., 1985), and the Quantitative Insulin Sensitivity Check Index (QUICKI) (Katz et al., 2000). We discovered that fasting glucose/insulin ratios were highly correlated to the HOMA‐IR (*r* = 0.85) and the QUICKI (*r* = 0.88). The fasting QUICKI showed an even better correlation to the HOMA‐IR (*r* = 0.94). Accordingly, we opted to use the QUICKI because it has been deemed “appropriate and effective for use in large epidemiological or clinical research studies and to follow changes after therapeutic interventions” (Patarrão et al., 2014). It is deemed “appropriate for use in studies where evaluation of insulin sensitivity is not of primary interest,” which, is the case for our healthy chronically active participants.

### 2.6 Cognitive assessment

On session 1, a subset of participants (12 younger adults and 17 older adults) completed two cognitive assessments using the National Institutes of Health (NIH) Toolbox: the dimensional change card sort (DCCS; RRID: SCR_003616) and the Flanker Inhibitory Control and Attention (Flanker; RRID: SCR_003617). These assessments have been thoroughly evaluated for reproducibility and reliability in the evaluation of attention and executive function (Carlozzi et al., 2014; Dikmen et al., 2014; Heaton et al., 2014; Tulsky et al., 2014; Zelazo et al., 2014). Seven participants opted not to complete the cognitive assessment. The DCCS assessment examines executive function by displaying a reference image to the participant prior to displaying two images where the participant must select the image that matches a specified criterion of either shape or color (Carlozzi et al., 2014; Zelazo, 2006). The Flanker assessment evaluated attention and executive function by displaying five arrows that point either left or right and has the user select the direction the middle arrow is pointing (Zelazo et al., 2014). Accuracy and time to select the answer are used to derive the final uncorrected score.

### 2.7 Statistical analysis

A studentized *T*‐Test was used to compare participant demographic and anthropometric variables between the younger and older adults. A mixed model repeated measures analysis of variance was used to compare changes in glucose, insulin, glucose‐insulin ratio, HOMA‐IR, QUICKI, and lactate during the standardized meal using a within‐group factor of condition (rest versus exercise) and a between‐group factor of age (younger versus older adult). Additionally, we also explored the role of biological sex as an additional factor in the mixed model repeated measures analysis of variance. Finally, we used regression analysis to examine associations among age, anthropometric assessments, venous biomarkers, and cognition assessments. Post hoc tests were completed using the Tukey procedure to correct for multiple comparisons where required. A significance level of 0.05 was used for all statistical significance testing. Results are presented as mean ± SD, unless otherwise stated.

## 3 RESULTS

Table 1 summarizes the demographic, anthropometric, physical activity levels, and baseline physiological measurements for the younger and older groups. There were no differences in height, weight, and body mass index (BMI) between older and younger adults, supporting the high level of fitness for the older group. Older adults had a higher body fat percentage and subsequently lower fat‐free mass (*p* < 0.001). The basal metabolic rate was significantly higher in younger adults compared to older adults (*p* = 0.04); however, the self‐reported physical activity level (PAL) was higher in older adults compared to younger adults (*p* < 0.001) so that the meal size was similar (*p* = 0.12). The older group had higher baseline HOMA‐IR (*p* < 0.04), QUICKI (*p* = 0.019), ECW/ICW ratios (*p* < 0.001), glucose (*p* < 0.001), and lower measures of cognitive function (*p* < 0.002).

### 3.1 Postprandial and physiologic responses to exercise

**FIGURE 1:**
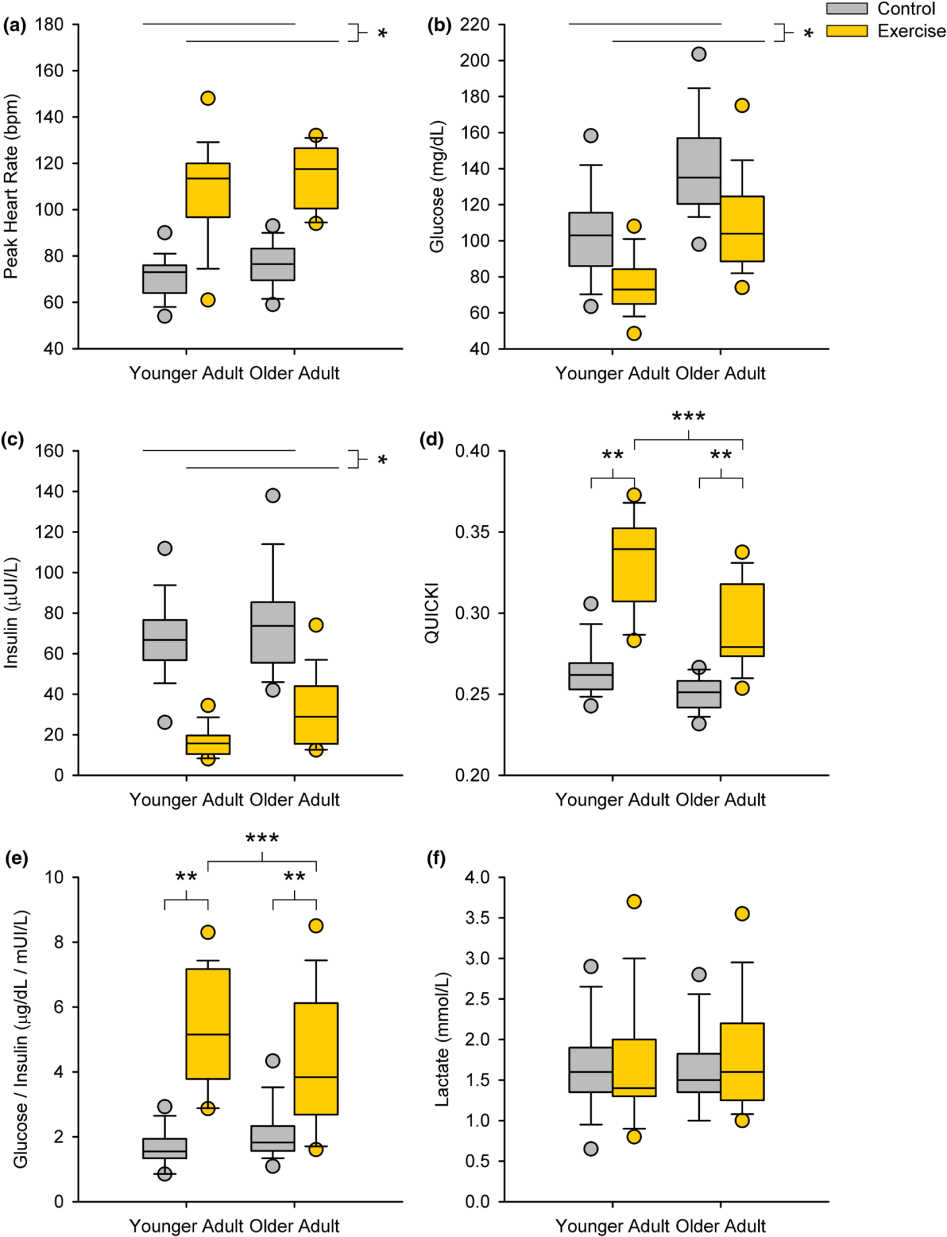
Standardized meal response. There was a significant (a) increase in peak heart rate (*p* < 0.001), (b) decrease in venous glucose (*p* < 0.001), (c) decrease in venous insulin (*p* < 0.001) after exercise compared to the control session with no significant interactions between the session and age (younger versus older adults) (*p* = 0.71, *p* = 0.53, and *p* = 0.18, respectively). There was a significant interaction between session and age for the (d) QUICKI (*p* < 0.001) and (e) glucose insulin ratio (*p* = 0.002). For both younger and older adults, the QUICKI (*p* < 0.001 and *p* = 0.003) and glucose insulin ratio (*p* < 0.001 and *p* = 0.003) were significant. Additionally, younger adults had a higher QUICKI (*p* < 0.001) and glucose insulin ratio (*p* = 0.002) after exercise, but there was no difference in the QUICKI (*p* = 0.24) or glucose insulin ratio (*p* = 0.40) after the control session. There were no significant differences in (f) lactate between the exercise and control sessions (*p* = 0.27) or between younger and older adults (*p* = 0.97). *Indicates a significant main effect between control (rest) and exercise session; **indicates a significant simple effect between control (rest) and exercise session within each age group; ***]indicates a significant simple effect between age groups within the exercise session.

The peak heart rates of younger and older adults were 71.5 ± 8.8 beats per minute (bpm) and 76.1 ± 9.2 bpm during the rest sessions. The peak heart rates of younger and older adults were 107.6 ± 20.8 bpm and 114.5 ± 13.2 bpm during the exercise sessions (Figure 1a). There was no interaction between session (exercise vs. rest) and age group (young vs. old) (*p* = 0.708) nor a main effect of age (*p* = 0.489). As expected, there was a significant increase in heart rate for the exercise sessions relative to the rest sessions (*p* < 0.001).

The postprandial glucose concentrations of younger and older adults were 102.2 ± 25.1 mg/dL and 140.3 ± 25.8 mg/dL during the control (rest) session, and 77.1 ± 17.4 mg/dL and 110.4 ± 27.2 mg/dL during the exercise session, respectively. The postprandial insulin concentrations of younger and older adults were 65.5 ± 19.2 μUI/mL and 72.0 ± 24.9 μUI/mL during the control (rest) session and 18.4 ± 10.7 μUI/mL and 36.1 ± 19.1 μUI/mL after the exercise sessions, respectively. The postprandial glucose to insulin ratios of younger and older adults were 1.7 ± 0.5 and 2.2 ± 0.2 during the control (rest) session, and 5.4 ± 2.5 and 3.7 ± 1.8 during the exercise session, respectively. The postprandial HOMA‐IR scores of younger and older adults were 17.6 ± 6.6 and 26.04 ± 10.2 during the control (rest) session, and 3.3 ± 2.1 and 8.9 ± 5.5 during the exercise session, respectively. The postprandial QUICKI scores of younger and older adults were 0.26 ± 0.02 and 0.25 ± 0.01 during the control (rest) session, and 0.33 ± 0.03 and 0.29 ± 0.03 during the exercise session, respectively. The postprandial lactate concentrations of younger and older adults were 1.7 ± 0.5 mmol/L and 1.6 ± 0.4 mmol/L during the control (rest) session and 1.7 ± 0.77 mmol/L and 1.8 ± 0.77 mmol/L during the exercise session.

There were no significant interactions found between age group (younger versus older) and condition (rest versus exercise) for venous glucose (*p* = 0.53), insulin (*p* = 0.18), HOMA‐IR (*p* = 0.30), and lactate (*p* = 0.75); but an interaction was found for the QUICKI (*p* < 0.001) and glucose‐insulin ratio (*p* = 0.002) at 30 min after the standardized meal. Venous glucose (Figure 1b), insulin (Figure 1c), and HOMA‐IR were lower after exercise relative to the rest condition (*p* < 0.001, all); however, there were no differences found for venous lactate (Figure 1f) concentration between rest and exercise conditions (*p* = 0.27). Venous glucose (Figure 1b), insulin (Figure 1c), and HOMA‐IR concentrations were lower for the younger adults compared to the older adults (*p* < 0.001, *p* = 0.030, and *p* < 0.001, respectively); however, lactate (Figure 1f) concentrations were not different between age groups (*p* = 0.97). The exercise condition had a larger QUICKI (Figure 1d) and glucose to insulin ratio (Figure 1e) for both the younger adults (*p* < 0.001, all) and older adults (*p* < 0.001 and *p* = 0.003). Further, younger adults had a larger QUICKI and glucose to insulin ratio during the exercise condition (*p* < 0.001 and *p* = 0.002), but the difference was not found during the rest condition (*p* = 0.24 and *p* = 0.40). Biological sex did not influence the major findings of the group analysis. We did discover that older adult males had higher insulin concentrations compared to younger adult males (*p* = 0.11) while older adult females had similar insulin concentrations compared to younger females (*p* = 0.99). No other biomarkers showed any differences between biological sex.

### 3.2 Association of anthropometrics and body water compartment on glucose and insulin

**FIGURE 2:**
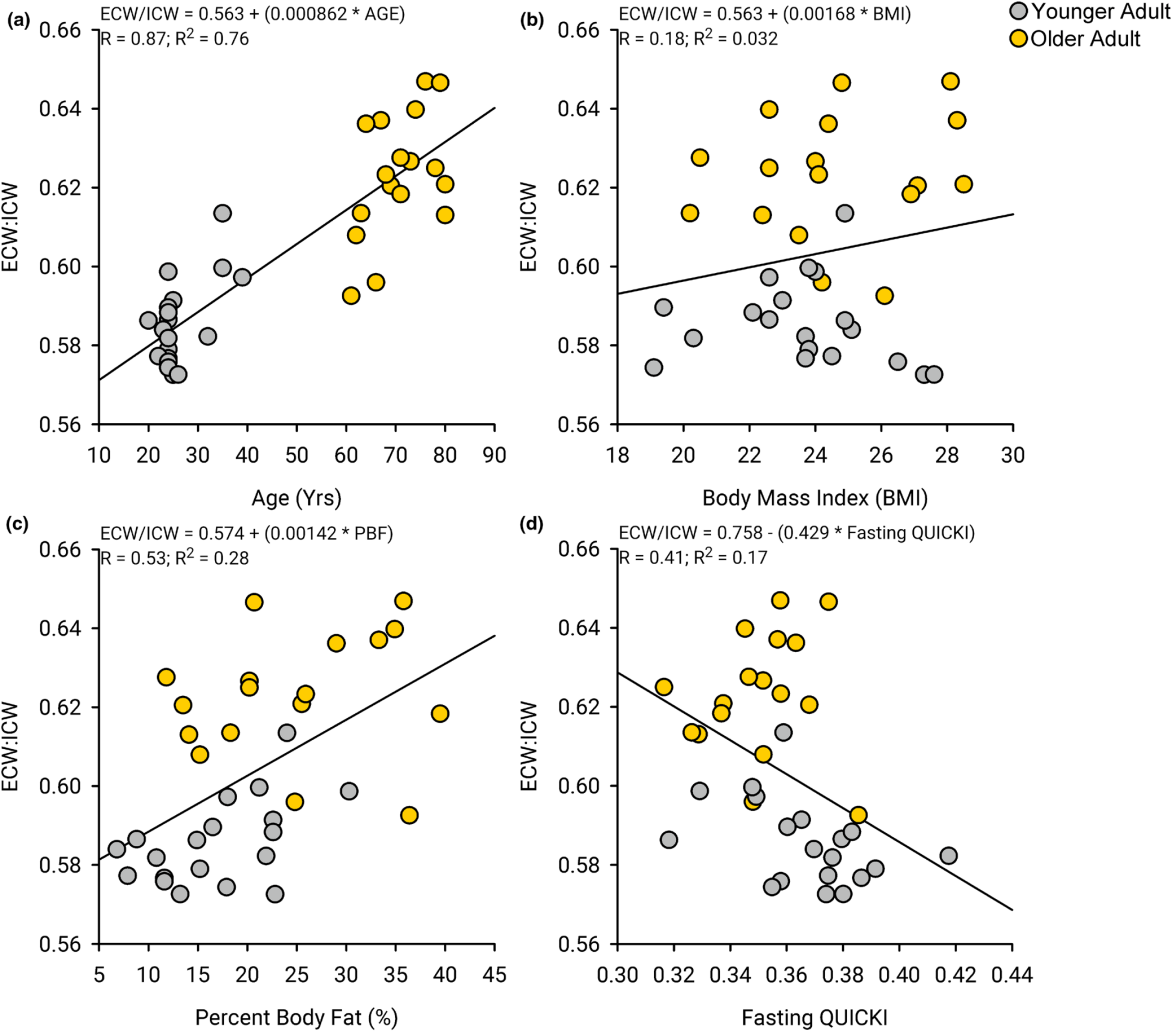
Correlations between age, body water compartment (ECW/ICW ratio), body mass index, and fasting metabolism (QUICKI). (a) There is a strong correlation between age and the ratio of extracellular water (ECW) to intracellular water (ICW) (*R* ^2^ = 0.756, *p* < 0.001). (b) There was no correlation found between the body mass index and the ratio of ECW to ICW and (*R* ^2^ = 0.032, *p* = 0.29). (c) There was a slight correlation found between percent body fat and the ratio of ECW to ICW and (*R* ^2^ = 0.28, *p* < 0.001). (d)There was a slight correlation found between the QUICKI and the ratio of ECW to ICW and (*R* ^2^ = 0.17, *p* = 0.013).

Age showed a strong correlation to the ratio of extracellular water to intracellular water (ECW/ICW) (*R* = 0.87, *R* ^2^ = 0.76, *p* < 0.001) (Figure 2a). There was no correlation found between the ECW/ICW ratio and height (*R* = 0.15, *R* ^2^ = 0.022, *p* = 0.39), weight (*R* = 0.02, *R* ^2^ = 0.0037, *p* = 0.91), body mass index (*R* = 0.18, R^2^ = 0.032, *p* = 0.29, Figure 2b), PAL (*R* = 0.27, *R* ^2^ = 0.071, *p* = 0.12), and BMR (*R* = 0.27, *R* ^2^ = 0.075, *p* = 0.11). There was a significant correlation found between percent body fat and the ECW/ICW ratio (*R* = 0.53, *R* ^2^ = 0.28, *p* < 0.001) (Figure 2c). There was a significant correlation found between the ECW/ICW ratio and fasting glucose (*R* = 0.39, *R* ^2^ = 0.15, *p* = 0.018) but not fasting insulin (*R* = 0.21, *R* ^2^ = 0.042, *p* = 0.23). There was a correlation found between the QUICKI and the ECW/ICW ratio (*R* = 0.41, *R* ^2^ = 0.17, *p* = 0.013, Figure 2d).

### 3.3 Body water compartment and change in QUICKI with exercise

**FIGURE 3:**
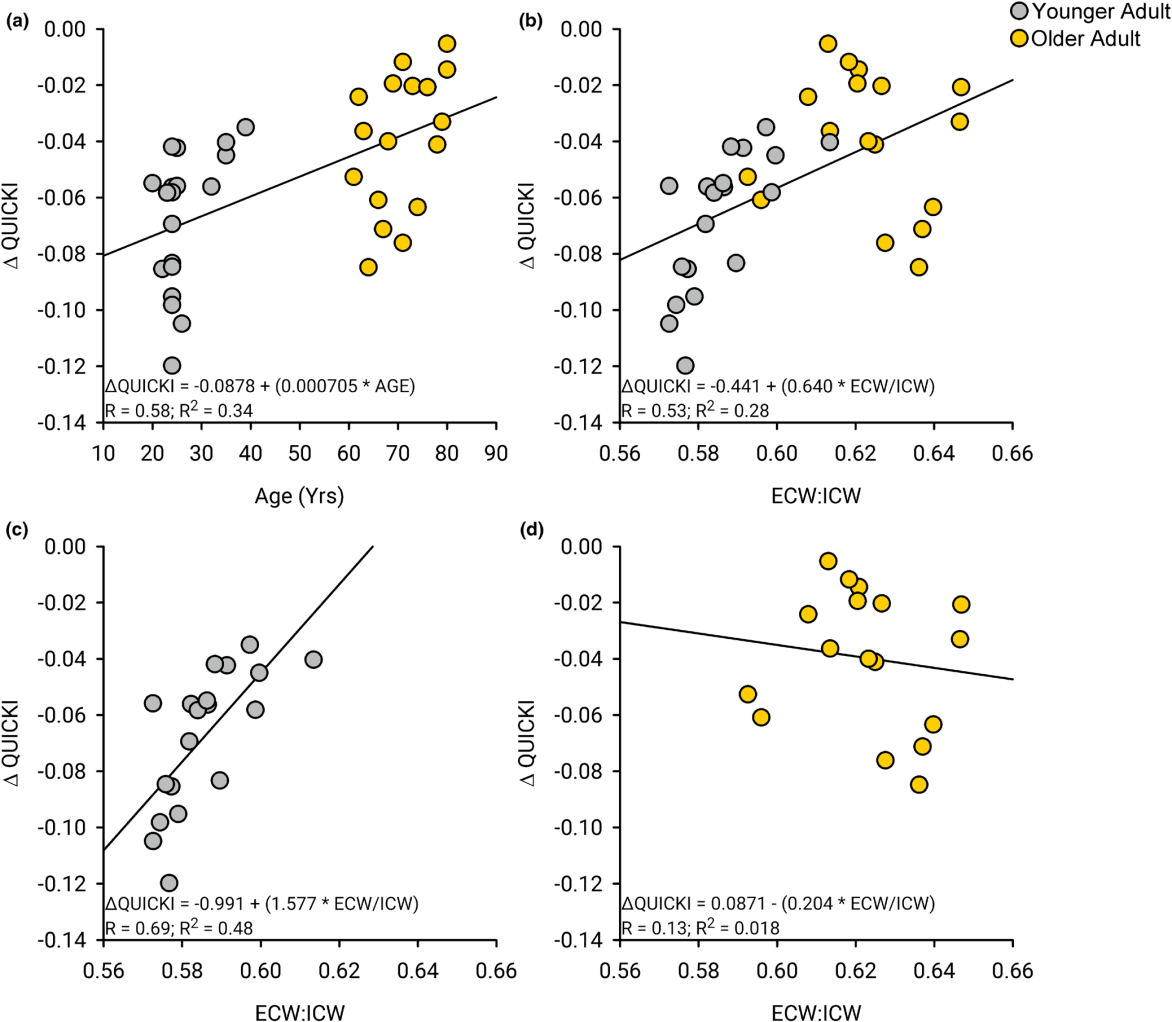
Body composition and change in QUICKI with exercise. (a) There was a moderate correlation between age and the difference in the QUICKI from the control session to the exercise session (*R* ^2^ = 0.34, *p* < 0.001). (b) There was a stronger correlation between the ratio of ECW to ICW and the difference in the QUICKI from the control session to the exercise session (*R* ^2^ = 0.28, *p* < 0.001). (c) When evaluating just the younger adult cohort, the correlation between the ratio of ECW to ICW and the QUICKI from the control session to the exercise session (*R* ^2^ = 0.48, *p* = 0.001). (d) When evaluating only the older adult cohort, the correlation between the ratio of ECW to ICW and the difference in the QUICKI from the control session to the exercise session (*R* ^2^ = 0.017, *p* = 0.62). This illustrates an improved sensitivity of these biomarkers for healthy and active younger adults compared to healthy and active older adults.

We found that chronological age was correlated to the change in QUICKI between the control and exercise sessions (*R* = 0.58, *R* ^2^ = 0.34, *p* < 0.001, Figure 3a). There was an even stronger correlation between the ECW/ICW and the change in QUICKI between the control and exercise sessions (*R* = 0.53, *R* ^2^ = 0.28, *p* < 0.001, Figure 3b). Separating by the younger and older adults, the correlation between the ECW/ICW and the change in QUICKI between the control and exercise sessions was stronger for the younger adults (*R* = 0.69, *R* ^2^ = 0.48, *p* = 0.001, Figure 3c) as compared to the older adults (*R* = 0.13, *R* ^2^ = 0.18, *p* = 0.093, Figure 3d). Hence, the range of ECW/ICW among younger adults was a more sensitive biomarker for evaluating metabolic health status compared to older adults. We estimated a sensitivity and specificity of 72% when using the 50th percentile values for the ECW/ICW. There were no significant correlations between ECW/ICW and the change in glucose (*R* = 0.052, *R* ^2^ = 0.0027, *p* < 0.76) and insulin (*R* = 0.24, *R* ^2^ = 0.056, *p* < 0.16) from the control session to the exercise sessions.

### 3.4 Cognitive function, body water compartment, and metabolic function

**FIGURE 4:**
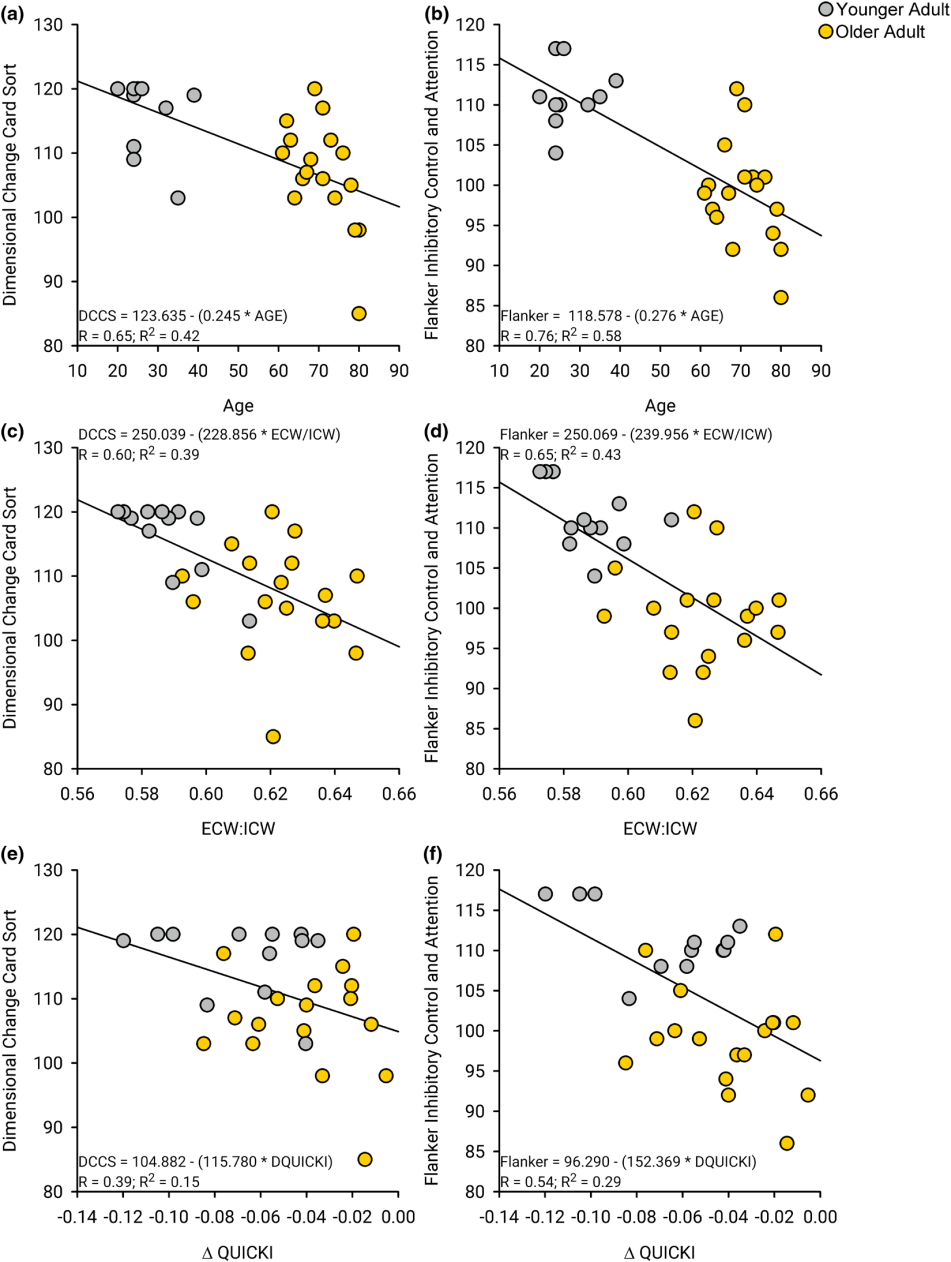
Body composition, change metabolic markers with exercise (QUICKI), and various assessments of cognitive function. (a) There was a moderate correlation between age and the dimensional change card sort cognitive assessment within the NIH Toolbox (*R* ^2^ = 0.42, *p* < 0.001). (b) There was a moderate correlation between age and the flanker inhibitory control and attention cognitive assessment within the NIH Toolbox (*R* ^2^ = 0.58, *p* < 0.001). (c) There was a moderate correlation between the ratio of ECW to ICW and the dimensional change card sort cognitive assessment within the NIH Toolbox (*R* ^2^ = 0.37, *p* < 0.001). (d) There was a moderate correlation between the ratio of ECW to ICW and the flanker inhibitory control and attention cognitive assessment within the NIH Toolbox (*R* ^2^ = 0.43, *p* < 0.001). (e) There was a moderate correlation between the difference in QUICKI from the control session to the exercise session and the dimensional change card sort cognitive assessment within the NIH Toolbox (*R* ^2^ = 0.15, *p* = 0.037). (f) There was a moderate correlation between the difference in QUICKI from the control session to the exercise session and the flanker inhibitory control and attention cognitive assessment within the NIH Toolbox (*R* ^2^ = 0.29, *p* = 0.003).

There was a significant difference between the performance of the younger and older adults during the DCCS and Flanker cognitive assessments (Table 1). The younger adults performed better on both cognitive assessments. There were strong correlations between chronological age and DCCS (*R* = 0.65, *R* ^2^ = 0.42, *p* < 0.001, Figure 4a), Flanker (*R* = 0.76, *R* ^2^ = 0.58, *p* < 0.001, Figure 4b), PSM (*R* = 0.67, *R* ^2^ = 0.45, *p* < 0.001), and LSM (*R* = 0.60, *R* ^2^ = 0.36, *p* < 0.0011) cognitive assessments. There were some correlations between fasting QUICKI and DCCS (*R* = 0.33, *R* ^2^ = 0.11, *p* = 0.08), Flanker (*R* = 0.38, *R* ^2^ = 0.15, *p* = 0.04), PSM (*R* = 0.39, *R* ^2^ = 0.15, *p* < 0.04), and LSM (*R* = 0.10, *R* ^2^ = 0.010, *p* = 0.61) cognitive assessments. However, there were strong correlations between performance on the cognitive assessments and the ECW/ICW and a change in QUICKI between the control and exercise sessions. Moderate correlations emerged between ECW/ICW and DCCS (*R* = 0.60, *R* ^2^ = 0.39, *p* < 0.001, Figure 4c), Flanker (*R* = 0.65, *R* ^2^ = 0.43, *p* < 0.001, Figure 4d), PSM (*R* = 0.49, *R* ^2^ = 0.24, *p* = 0.007), and LSM (*R* = 0.47, *R* ^2^ = 0.22, *p* < 0.0011) cognitive assessments. Lastly, there were correlations between the change in QUICKI between the control and exercise sessions and DCCS (*R* = 0.39, *R* ^2^ = 0.15, *p* = 0.04, Figure 4e), Flanker (*R* = 0.54, *R* ^2^ = 0.29, *p* = 0.003, Figure 4f), PSM (*R* = 0.34, *R* ^2^ = 0.12, *p* = 0.07), and LSM (*R* = 0.33, *R* ^2^ = 0.11, *p* = 0.08) cognitive assessments.

For those that performed the cognitive assessments, the variance in the postprandial change in QUICKI was largely explained by age (*R* = 0.55; *R* ^2^ = 0.31, *p* = 0.002). When both age and cognitive score (Flanker) were used to predict the change in QUICKI with exercise (*R* = 0.59; *R* ^2^ = 0.34, *p* = 0.005) the model explained ~4% more of the variance in the QUICKI.

## 4 DISCUSSION

We determined the effect of mild exercise on postprandial glucose and insulin among a healthy group of younger and older adults and examined the relationships between post‐prandial metabolic response, body water compartments (ECW/ICW), and cognitive function. After 15 min of walking, post‐prandial blood glucose levels decreased by ~20% in older and younger adults, while insulin concentrations decreased 50% and 70% in older and younger adults, respectively. Fasting glucose and insulin were not correlated with either ECW/ICW or cognitive function. However, the magnitude of change in the QUICKI with postprandial exercise was correlated to ECW/ICW and cognitive function, and was more sensitive in younger adults than older adults. Taken together, we showed, for the first time, a robust therapeutic potential of mild postprandial exercise on glucose and insulin in a head‐to‐head comparison among younger and older adults, and demonstrated, univariate relationships between postprandial glucose and insulin exercise responses, ECW/ICW body water ratios, and cognitive function in younger and older adults.

We confirmed that ambulation after a meal lowers postprandial hyperinsulinemia (DiPietro et al., 2013). Mechanistically, a meal initiates a transient state of hyperglycemia and subsequent hyperinsulinemia. When people remain sedentary, pancreatic insulin secretion increases and triggers hepatic, muscle, and adipose cells to uptake glucose through well‐defined transport mechanisms (Zierath, 1995; Zierath et al., 1996). Uniquely, muscle cells that are not “active” have a more limited capacity to uptake glucose in response to a meal. Postprandial muscle activity is thought to capitalize on the insulin‐independent AMPK pathway to move glucose into the skeletal muscle (Janssen & Hopman, 2003; Wasserman & Ayala, 2005). Because the walking intensity and duration were mild (60% maximal HR; 15 min) for the healthy people in this study, we expected that primarily oxidative skeletal muscle would be recruited for the task, minimizing any buildup of lactate, which was confirmed in a subset of the data (unpublished). While glycolytic muscle also utilizes the insulin‐independent pathway during bouts of activity (Blaauw et al., 2013; Schnyder & Handschin, 2015; Zampieri et al., 2015) the level 4 on the Borg Scale supported that the 15 min of walking was primarily oxidative. Accordingly, postprandial exercise at a mild level for only 15 min may be a “first line” preventative lifestyle recommendation for younger and older healthy adults to avoid glucose and insulin spikes after a meal, even among people who are active exercisers. The extent to which these findings are applicable to people with significant metabolic pathology warrants careful evaluation in future studies.

We also confirmed that the differences in body water compartments with age (Ekingen et al., 2022; Lee & Shields, 2022; Tuuri et al., 2005) that were associated with cognitive function (Lee & Shields, 2022), were also associated with the post‐prandial change in glucose/insulin ratios. We proposed that an individual's postprandial responsiveness to exercise may also be associated with cognitive function and secondarily with the distribution of body water, particularly across age. The theoretical basis for this hypothesis was that segmental bioimpedance studies show that carbohydrate ingestion increases muscle glycogen and is accompanied by a rise in ICW, but not ECW (Shiose et al., 2016). The notion that muscle‐derived AMPK‐activated Glut‐4 translocation may be regulated by changes in glycogen levels during post‐prandial exercise is intriguing as it forms a link between ECW/ICW, metabolism, and cognitive function (Richter et al., 2025).

While we discovered significant associations between glucose/insulin postprandial exercise and ECW/ICW body water ratio and cognitive function, we are cautious not to over‐interpret these early findings. The congruence observed between the capacity to influence postprandial glucose–insulin with exercise and cognitive function among healthy people is an interesting association, but more detailed experimentation needs to proceed to clearly understand the underlying mechanisms driving this association. The variance in the post‐prandial glucose–insulin response among healthy younger and older adults, which was associated with cognitive function and impaired ECW/ICW body water ratios needs further study. For example, future studies need to manipulate ECW/ICW and glycogen levels, then assess if there are changes in post prandial exercise metabolites and cognitive function (Michou et al., 2023).

To our knowledge, this is the first study to examine the glucose/insulin postprandial response to exercise and associate it with cognitive function and extracellular/intracellular body water. Low and colleagues should be credited with this initial idea when they showed that extracellular water was associated with attention and memory function scaled among people with various levels of diabetes (Bellini, Nicolo, Bazzucchi, & Sacchetti, 2022). Details of their paper were insightful as they showed that extracellular water, as an index of edema, was aligned with pulse pressure, hypertension, and glycemic control (Bellini, Nicolo, Bazzucchi, & Sacchetti, 2022). Leakage of blood plasma through the blood–brain barrier as a result of high blood pressure is believed to contribute to even mild cognitive impairment (Li et al., 2021). Hypertension may damage blood vessels, leading to vessel stiffness leading to hypometabolism to the brain (Li et al., 2021). While the exact mechanisms remain elusive, the cognitive tests for executive function, inhibition, and memory were lower among those who had the poorest response to postprandial glucose‐insulin with exercise; and also showed higher ECW/ICW body water ratios. Future research is needed to better understand the utility of ECW/ICW, metabolic response to exercise, and onset of cognitive impairment. Timely exercise, after each meal, at the correct dose, and over a lifetime may form the underlying basis to support a lifestyle needed to mitigate the escalating prevalence of early dementia and metabolic disease.

### 4.1 Limitations and future direction

This study was adequately powered for the detection of acute change in postprandial insulin regulation during a standardized meal, but a larger sample including people with a greater range of cognitive function would improve our ability to understand the relationships examined in this study. Second, venous blood samples were obtained at two distinct time points providing a snapshot of the metabolic response. Obtaining more blood sampling points throughout each session would provide a more detailed assessment of the physiologic responses to our postprandial exercise. Finally, a larger sample size would enable us to assess people from various backgrounds and demographics and tease out the co‐variates known to be associated with age that also likely influence metabolic flexibility and cognitive function. Our goal is to develop interventions that are affordable and that can be tailored for each person, especially those from rural communities that are at a higher risk of developing metabolic disease.

## 5 SUMMARY AND CONCLUSIONS

Exercise is underutilized by the healthcare system in the United States (Petrie et al., 2023). While the barriers to exercise are complex and multifactorial, we believe that we can improve exercise prescription compliance by developing a strong alliance with medical providers to better understand why exercise may be effective. Sound exercise prescriptions need to be precise for each health condition and grounded in sound physiological principles. This study demonstrates that 15 min of low‐to‐moderate intensity walking after a meal improves blood glucose–insulin regulation in active healthy younger and older groups, a finding unique to this study. Interestingly, we discovered that cognitive function was moderately correlated to the change observed in the postprandial glucose–insulin response and also associated with body water compartments (ECW/ICW). Future studies are warranted to develop a multivariate model to predict cognitive impairment based on metabolic biomarkers.

## AUTHOR CONTRIBUTIONS

RKS conceived and designed this research study. MAP, KAJ, PMM, AA, TB, KG, JH, AK, JK, ML, MP, and RKS performed experiments, analyzed data, and interpreted results of experiments. MAP, KAJ, PMM, AA, and RKS prepared figures and drafted the manuscript. MAP, KAJ, PMM, AA, TB, KG, JH, AK, JK, ML, MP, and RKS edited, revised, and approved the final version of the manuscript.

## FUNDING INFORMATION

This study was supported in part by awards to RKS from the National Institute of Child Health and Human Development Grant R01HD084645 and R01HD082109.

## CONFLICT OF INTEREST STATEMENT

The authors have no perceived or potential conflict of interest financial or otherwise.

## ETHICS STATEMENT

All participants provided written informed consent. This study was conducted in accordance with the Declaration of Helsinki and received approval from the Internal Review Board (IRB) at the University of Iowa (IRB: 202104727).
